# Universal Character of Breaking of Wormlike Surfactant Micelles by Additives of Different Hydrophobicity

**DOI:** 10.3390/nano12244445

**Published:** 2022-12-14

**Authors:** Andrey V. Shibaev, Alexander S. Ospennikov, Elizaveta K. Kuznetsova, Alexander I. Kuklin, Teimur M. Aliev, Valentin V. Novikov, Olga E. Philippova

**Affiliations:** 1 Physics Department, Moscow State University, 119991 Moscow, Russia; 2 Frank Laboratory of Neutron Physics, Joint Institute for Nuclear Research, 141980 Dubna, Russia; 3 Moscow Institute of Physics and Technology, 141701 Dolgoprudny, Russia; 4 A. N. Nesmeyanov Institute of Organoelement Compounds, Russian Academy of Sciences, 119991 Moscow, Russia

**Keywords:** wormlike surfactant micelles, viscosity, solubilization, diffusion-ordered NMR spectroscopy (DOSY)

## Abstract

Wormlike surfactant micelles are widely used in various applications including fracturing technology in oil industry, template synthesis of different nanoobjects, micellar copolymerization of hydrophilic and hydrophobic monomers, and so forth. Most of those applications suggest the solubilization of different additives in the micelles. The present paper is aimed at the comparative study of the effect of the solubilization of hydrophobic (n-decane and 1-phenylhexane) and hydrophilic (N-isopropylacrylamide and acrylamide) substances on the rheological properties and structure of the micelles using several complementary techniques including rheometry, small angle neutron scattering, dynamic light scattering, and diffusion ordered NMR spectroscopy. For these studies, mixed micelles of potassium oleate and n-octyltrimethylammonium bromide containing the excess of either anionic or cationic surfactants were used. It was shown that hydrophobic additives are completely solubilized inside the micelles being localized deep in the core (n-decane, 1-phenylhexane) or near the core/corona interface (1-phenylhexane). At the same time, only a small fraction of hydrophilic additives (14% of N-isopropylacrylamide and 4% of acrylamide) penetrate the micelles being localized at the corona area. Despite different localization of the additives inside the micelles, all of them induce the breaking of wormlike micelles with the formation of either ellipsoidal microemulsion droplets (in the case of hydrophobic additives) or ellipsoidal surfactant micelles (in the case of hydrophilic additives). The breaking of micelles results in the drop of viscosity of the solution up to water value. The main result of this paper consists in the observation of the fact that for all the additives under study, the dependences of the viscosity on the volume fraction of additive lie on the same master curve being shifted along the volume fraction axis by a certain factor depending on the hydrophobicity of the added species. Those data are quite useful for various applications of wormlike surfactant micelles suggesting the solubilization of different additives inside them.

## 1. Introduction

Many surfactants are able to self-assemble spontaneously into very long cylindrical aggregates—wormlike micelles (WLMs) [1,2,3,4]. The length of micellar chains can reach several micrometers. Like polymeric chains, the WLMs can entangle with each other, which imparts viscoelastic properties to the solutions [3,5,6,7,8]. The rheological behavior of WLMs can be described by “living polymer” models that account for breakage and recombination of the micelles [9,10,11].

The micellar chains can accommodate various substances in their interior. Being of amphiphilic nature, the WLMs solubilize both hydrophobic [12,13,14,15,16,17,18,19,20,21,22] and hydrophilic [23,24] substances. Most of the hydrophobic substances are preferentially solubilized in the micellar core, whereas the hydrophilic substances prefer to reside in the corona area.

Since the WLMs represent a non-covalent assembly, the solubilization often changes the structure of the micelles. For instance, the solubilization of aliphatic hydrocarbons in the hydrophobic core results in the transformation of WLMs into small microemulsion droplets [18,19,21]. This property is exploited in fracturing technology in oil industry for the creation of artificial media with high permeability with respect to oil [25,26,27,28,29,30,31]. Those media are based on the suspension of proppant (sand or ceramic) particles in WLM solution. In the absence of oil, the entangled WLMs create a highly viscous medium preventing the sedimentation of proppant particles. In the presence of oil, the WLMs are destroyed, producing low viscous medium, which does not hinder the flow of oil. Note that some aromatic hydrocarbons may be solubilized near the core/water interface (in particular, in cationic surfactant micelles), which increases the viscosity [13]. The enhancement of viscosity is attributed to the increase in the micellar length because of the screening of the electrostatic repulsion between the surfactant headgroups and the lowering of the free energy of hydrocarbon/water interface [12,32]. However, when the outer layer becomes saturated with aromatic hydrocarbon, newly added hydrocarbon molecules penetrate deep inside the core, which finally results in the desired effect—a decrease in viscosity [13].

The solubilization ability of WLMs is also exploited in various modern applications, which include the use of WLMs as nanoreactors [33,34], as a template for directed growth of inorganic nanoparticles [35,36] and nanochains [37] or for polymerization of water-insoluble monomers [38]. In particular, solutions of WLMs can be utilized for conducting micellar copolymerization of a water-soluble and a water-insoluble comonomers. Those reactions start in water, but when the growing radical meets a micelle, all hydrophobic monomers residing in the micelle incorporate into the polymer chain [39,40,41,42,43]. As a result, a hydrophobic block is formed in the growing macromolecule. Currently, mainly spherical micelles are used in micellar copolymerization [39,40]. Those micelles can solubilize only a rather small amount of hydrophobic monomer, thereby providing a low degree of blockiness of the synthesized copolymer [40]. At the same time, a high degree of blockiness is required to obtain tough hydrogels through association of hydrophobic blocks of different polymer chains [44]. High degree of blockiness can be achieved when long WLMs are used instead of small spherical ones to solubilize the hydrophobic monomer [45,46]. For instance, in this way, very tough gels were prepared from a multiblock copolymer of acrylamide (AA, water-soluble hydrophilic monomer) and n-alkylacrylates (hydrophobic monomers) [45,46]. Later, the micellar copolymerization in WLM solution was used to synthesize double networks [47,48] and nanocomposite networks [49]. Note that the prepared gels were not only very tough, but also self-healable, and highly stretchable because of considerable strength of cross-links combined with their ability to reversibly break and restore.

Since the key factor providing high blockiness of the synthesized copolymer is a large amount of hydrophobic monomer which can be absorbed by WLMs [23], most of the studies were devoted to the solubilization of hydrophobic substances in WLMs [19,20,21,22]. Much less attention was paid to the effect of water-soluble monomers on the WLMs, even though their concentrations are always by two orders of magnitude higher than those of the hydrophobic monomer [24]. At the same time, water-soluble monomer may also influence the properties of the micellar aggregates. For instance, it was shown that AA can induce a shortening of WLMs [24] as well as the transition from short cylindrical to spherical micelles [23]. AA can also affect the spherical SDS micelles [43] by reducing their aggregation number or even preventing micellization, which was explained by the presence of a rather hydrophobic C=C double bond in the monomer, which causes its incorporation into the micelle. To the best of our knowledge, the effect of other widespread hydrophilic monomers on the WLMs was not studied.

Thus, both hydrophobic and hydrophilic additives solubilizing within the WLMs finally lead to the disruption of long micelles, which results in the decrease in the viscosity of surfactant solution. However, no comparison of different hydrophobic and hydrophilic additives on the structure of micellar aggregates and the viscosity of surfactant solution has been performed. At the same time, this knowledge is important for further elaboration of synthetic strategy for preparing nanostructures using the micelles as a nanoreactor or a template.

The aim of the present paper is to compare the effects of additives with different hydrophobicity on the structure of micellar aggregates and the viscosity of their solution. For this purpose, four additives were used including water-insoluble hydrocarbons (n-decane and 1-phenylhexane) and water-soluble monomers (n-isopropylacrylamide (NIPA) and AA). The experiments were performed with mixed micelles of potassium oleate and n-octyltrimethylammonium bromide (C8TAB) at two C8TAB/oleate ratios corresponding to the excess of either anionic or cationic surfactant. It was observed that despite the different hydrophobicity of the additives and their different location inside the surfactant aggregates, the dependences of the viscosities of surfactant solutions on the concentration of added species lie on the same universal master curve upon shift along the concentration axis. The reasons behind this universal behavior are discussed.

## 2. Materials and Methods

### 2.1. Materials

Potassium oleate (TCI, Tokyo, Japan, >98%), C8TAB (J&K Scientific, Beijing, China, >98%), n-decane (Sigma Aldrich, St. Louis, MO, USA, >99%), 1-phenylhexane (Sigma Aldrich, St. Louis, MO, USA, >99%), NIPA (Sigma Aldrich, St. Louis, MO, USA, >98%), AA (Sigma-Aldrich, St. Louis, MO, USA, >98%), and potassium hydroxide (Acros, Geel, Belgium, >98%) were used as received. Aqueous solutions were prepared with distilled deionized water obtained with Milli-Q Water Purification System (Millipore, Burlington, MA, US). D_2_O (AstraChem, Saint-Petersburg, Russia, 99.9% isotopic purity) was utilized for the solutions for SANS and NMR studies.

### 2.2. Samples Preparation

First, stock solutions of two surfactants (C8TAB and potassium oleate) in water were prepared, and their pH was adjusted to 11.0 ± 0.1 with an appropriate amount of potassium hydroxide solution. The stock solutions were mixed in required quantities to obtain the desired concentrations of the surfactants (10^−3^ M KOH was used as a solvent). Then, different quantities of additives were added, and the resulting system was stirred for one night with a magnetic stirrer and then left to equilibrate for few days.

### 2.3. Rheometry

The rheological measurements were performed with a stress-controlled rotational rheometer Physica SmartPave 102 (Anton Paar, Graz, Austria) as described elsewhere [35,36]. The viscous samples (with zero-shear viscosity η_0_ > 0.01 Pa·s) were studied using cone-plate geometry (diameter 40 mm, cone angle 2°) with a cell cover for preventing solvent evaporation. The less viscous samples (η_0_ < 0.01 Pa·s) were measured in double-gap cylindrical geometry (mean diameter 26.4 mm, height 40 mm, gap 0.42 mm). In all experiments, the temperature was controlled with high precision (20.00 ± 0.05 °C) using Peltier elements. In steady shear experiments, the shear rate was changed from 0.005 to 10 s^−1^. The zero-shear viscosity was determined from the viscosity at the low-shear-rate plateau. In oscillatory shear experiments, the angular frequency ω was varied from 0.006 to 300 s^−1^. All measurements were made in a linear viscoelastic regime, which had been preliminarily determined by the deformation amplitude sweep measurements at ω = 10 s^−1^, so that both dynamic moduli (the storage G’ modulus and the loss G” modulus) did not depend on the deformation amplitude.

### 2.4. SANS

Small-angle neutron scattering (SANS) experiments were carried out on the YuMO spectrometer with two ring detectors that cover the dynamical range of scattering vectors (q) from 0.005 to 0.55 Å^−1^. The spectrometer is located at the IBR-2 pulsed reactor of the Frank Laboratory of Neutron Physics (Joint Institute for Nuclear Research, Dubna, Russia). For the measurements, the specimens were put in Hellma quartz cells (thickness—2 mm). The raw SANS data were corrected for the sample transmission, sample thickness and electronic noise by SAS program [50,51,52]. Scattering of KOH solution in D_2_O at pD 11.4 was subtracted from the data as the background scattering. For fitting of the scattering curves, the program SasView was used. The curves were fitted by models of a cylinder with elliptical cross-section or of a charged prolate ellipsoid. In the case of charged ellipsoids, Hayter–Penfold Rescaled Mean Spherical Approximation (RMSA) structure factor was employed, and the following fitting parameters were used: equatorial R_eq_ and polar R_pol_ radii, their polydispersities, and ellipsoid charge (in electrons). In the case of an elliptical cylinder, the following fitting parameters were used: radii of the elliptical cross-section R_1_ and R_2_, and their polydispersities. The fitting was performed at q > 0.05 Å^−1^, where the influence of interactions is negligible, and then the fit was extrapolated to the low-q range. SANS experiments were conducted in a pinhole geometry, and instrumental resolution of dq/q = 0.05 was included in the fitting procedure.

### 2.5. DLS

Dynamic light scattering (DLS) measurements were made on an ALV/DLS/SLS-5000 goniometer system (Langen, Germany) equipped with an ALV-5000 digital time correlator and a helium–neon laser operating at a wavelength of 632.8 nm. The data treatment was performed as described elsewhere [53,54]. Before the measurements, the solutions were filtered through 0.22 μm polyvinylidenefluoride filters (Millipore) and poured into cylindrical cells (10 mm in diameter). The cells were immersed in toluene, the refractive index matching fluid. The distributions of decay times were determined from the measured autocorrelation functions of the scattered intensity by using a CONTIN method [55].

### 2.6. DOSY NMR

Diffusion ordered spectroscopy (DOSY) measurements were carried out on a Bruker Avance 600 spectrometer using a DOSY-ONESHOT pulse sequence [56] with the following parameters: 0.2 s diffusion time and 1 ms gradient pulse duration for determination of self-diffusion coefficients of additive molecules; 0.6 s diffusion time and 2.5 ms gradient pulse duration for micelles; 5 s relaxation delay; unbalancing factor α = 2. To achieve signal attenuation, the gradient strength was increased from 5% to 80% as defined by the pulse sequence in 64 steps, with 16 scans each with a maximum gradient strength of 0.27 T·m^−1^. The rows of the pseudo-2D diffusion dataset were phased and baseline corrected. The diffusion coefficients were determined using MestReNova software (version 14.2.1-27684, MestReLab Research S.L., Santiago de Compostela, Spain).

## 3. Results and Discussion

In this work, we systematically investigate the effect of additives with different hydrophobicity on WLMs formed by a mixture of an anionic (potassium oleate) and cationic (C8TAB) surfactants. The concentration of potassium oleate is fixed at 2.5 wt% (78 mM), and we use two concentrations of C8TAB: 0.98 and 2.95 wt% (39 and 117 mM), which correspond to the molar ratios R = [C8TAB]/[oleate] equal to 0.5 and 1.5. Therefore, the studies were performed both at an excess of anionic (R = 0.5) or cationic (R = 1.5) surfactant. We used several additives—two water-soluble monomers: more hydrophilic acrylamide (AA, solubility in water 2155 g/L at 30 °C [57]) and less hydrophilic N-isopropylacrylamide (NIPA, solubility in water 212 g/L at 20 °C [58]), and two water-insoluble hydrocarbons: 1-phenylhexane (solubility in water 10^−3^ g/L) and n-decane (solubility in water 9·10^−6^ g/L [59]). The first hydrocarbon (1-phenylhexane) contained an aromatic ring, the second hydrocarbon (n-decane) was purely aliphatic.

### 3.1. Overall Effect of Additives on Viscoelastic Properties

**Figure 1:**
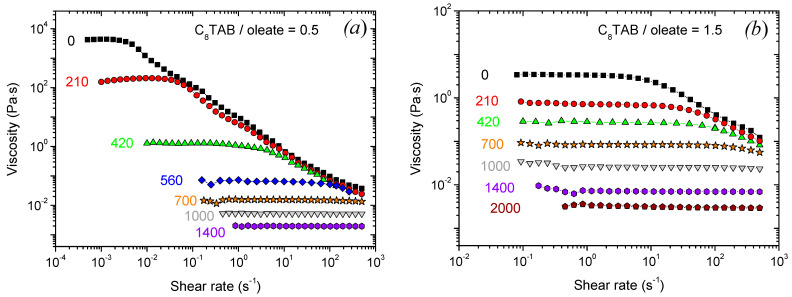

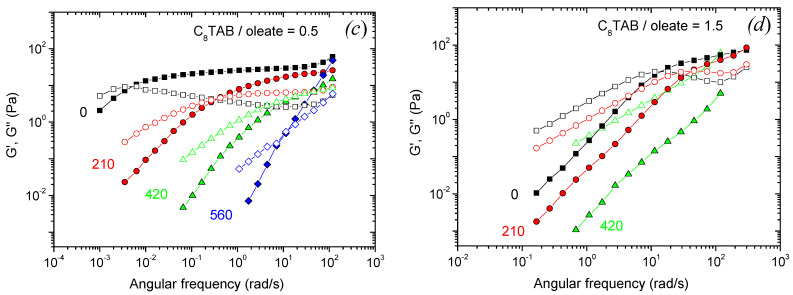
(**a**,**b**) Flow curves, (**c**,**d**) frequency dependences of the storage G’ (filled symbols) and loss moduli G” (open symbols) for aqueous solutions containing 78 mM potassium oleate, 39 mM (**a**,**c**) or 117 mM (**b**,**d**) C8TAB and different amounts of AA indicated in the Figure in mM. Temperature: 20 °C, pH 11.

First, let us treat the effect of various additives on viscoelastic properties of C8TAB/oleate solutions. Note that at chosen concentrations of the surfactants, in the absence of additives, the networks of mixed WLMs are formed at both molar ratios of two surfactants R = 0.5 [19] or 1.5 [30]. This is manifested by high viscosities which are 3–6 orders of magnitude higher than the viscosity of water (4400 Pa·s at R = 0.5 and 3.5 Pa·s at R = 1.5) (Figure 1a,b) and by pronounced viscoelastic properties (Figure 1c,d).

A general effect on viscoelastic properties is similar for all the additives and for both surfactant molar ratios R, and is represented in Figure 1 (AA), Figure S1 (NIPA), Figure S2 (1-phenylhexane) and in [19,30] (n-decane). When the additive concentration is increased, the zero-shear viscosity drops down to the values close to water (~10^−3^ Pa·s). This is accompanied by a disappearance of shear-thinning behavior and viscoelasticity: (i) high-frequency elastic plateau at the dependence G’ (ω) becomes narrower and diminishes; (ii) the cross-over point between G’ (ω) and G” (ω) moves to higher frequencies, meaning a drop of the characteristic relaxation time; (iii) G’ (ω) and G” (ω) curves progressively transform from those characteristic of a viscoelastic fluid to those inherent to a Newtonian liquid. Similar effects are observed for the addition of hydrocarbons to various WLMs [12,17,18,19] as well as for the addition of acrylamide to WLMs formed by a single surfactant potassium oleate [24]. This behavior is explained by breaking of the WLMs network as a result of additive solubilization inside the micelles. In the present paper, we first compare the effect of additives with different hydrophobicity on the WLMs.

**Figure 2:**
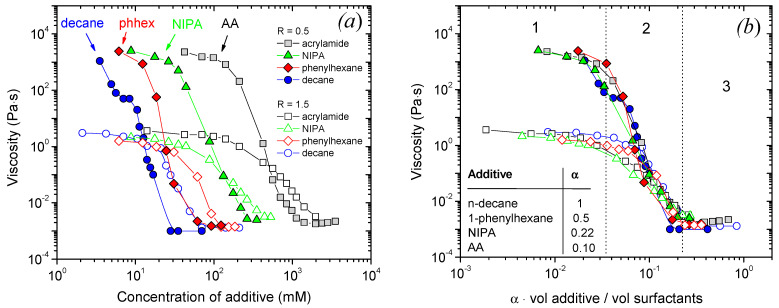
Dependences of zero-shear viscosity on additive concentration (**a**) or on volume ratio α·(vol. additive)/(vol. surfactant) (**b**) for aqueous solutions containing 78 mM potassium oleate, 39 mM (filled symbols) or 117 mM (open symbols) C8TAB and different additives: acrylamide AA (squares), n-isopropylacrylamide NIPA (triangles), 1-phenylhexane (diamonds), n-decane (circles). Temperature: 200C, pH 11. In Figure 2a, the curves for n-decane are reproduced with permission from [19,30].

In order to compare the influence of all additives on WLMs, the dependences of zero-shear viscosity on additive concentration are summarized in Figure 2a. It can be seen that the curves generally have a similar shape: the viscosity decreases and reaches the viscosity of water. However, the curves are shifted in the horizontal direction when the hydrophobicity of the additive decreases, that is, the lower the hydrophobicity, the larger the shift: n-decane → 1-phenylhexane → NIPA → AA. Predictably, higher concentrations of less hydrophobic molecules are necessary to induce the drop of viscosity and further transformation to a liquid with viscosity close to water value. A similar effect was previously observed for WLMs formed by a cationic surfactant erucyl-bis-hydroxyethylmethylammonium bromide: a much higher concentration of ethanol (1302 mM) than that of n-decane (93 mM) was required to induce a drop of viscosity from 1000 to 20–50 Pa·s [60]. However, in [60], only very small concentrations of the additives were studied, so that the WLM networks were not broken. In the present work, a wide concentration range is studied, so that we can observe a whole course of the progressive decrease in the viscosity.

**Figure 3:**
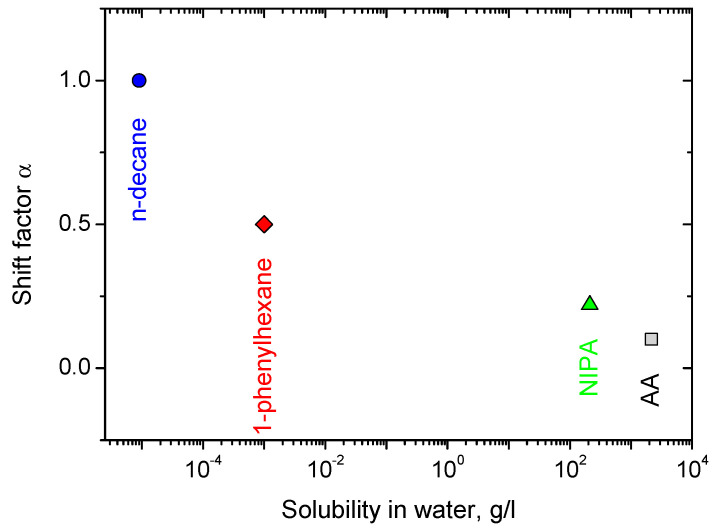
Dependence of the shift factor of the viscosity α on the solubility of the additive in water.

Figure 2b shows that all the viscosity dependences can be superimposed on a “master” curve by shifting them along the additive concentration axis. This may be done by dividing the additive volume by the total volume of two surfactants (which is different for R = 0.5 and 1.5 due to different amounts of C8TAB molecules), and then multiplying this relative volume of additive by a factor α, which is taken equal to unity for the most hydrophobic n-decane, and is less than one for the other additives. Here, the total volume of the surfactants was calculated as a sum of dry volumes of oleate and C8TAB, e.g., masses of added surfactants divided by their densities. The factor α may be treated as an “efficacy” of a certain additive in disrupting the WLMs. The α value has the following semi-quantitative meaning: if some n-decane concentration C_dec_ induces a certain decrease in viscosity, then C_dec_/α is the concentration of the other additives which results in the same drop in viscosity. The α value is correlated with the hydrophobicity of the additive: e.g., α decreases with an increase in the additive solubility in water (Figure 3).

If molar ratios [additive]/[surfactants] are used instead of the volume ratios, all the curves may also be superimposed (Figure S3a), and their general view does not differ from the case of volume ratios. However, we suppose that it is better to use volume ratios in order to characterize which part of the volume inside the surfactant aggregates is occupied by additive molecules.

Note that hydrocarbons and monomers are expected to have a different effect on WLMs. Indeed, highly hydrophobic molecules are usually solubilized deep inside the micellar hydrophobic cores [18], while acrylamide was shown to reside closer to the micellar corona [24]. Surprisingly, the superimposition works perfectly well both for two hydrocarbons, which are almost insoluble in water, and for two water-soluble monomers, and results in a good overlapping of the curves for all four additives. Although the whole course of WLMs disruption is similar for all the additives, the only principal difference being higher initial viscosity for R = 0.5 than for R = 1.5.

Therefore, all the additives act similarly on WLMs, though higher concentrations of less hydrophobic additives are necessary to induce the viscosity drop. To understand the origins of this effect, detailed studies of the WLMs disruption by each additive were performed, which are described below.

### 3.2. Correlation of the Evolution of Viscosity and Structural Changes

**Figure 4:**
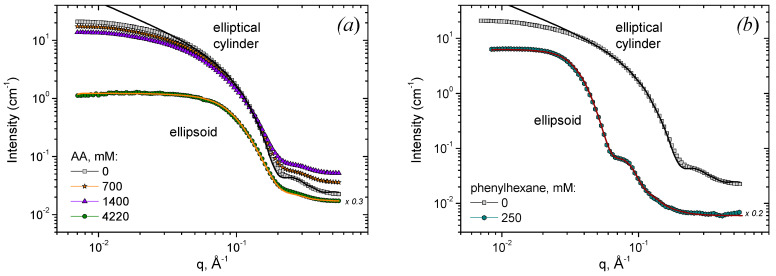
SANS scattering curves for aqueous solutions containing 78 mM potassium oleate, 117 mM C8TAB and different concentrations of AA (**a**) or 1-phenylhexane (**b**) indicated in the Figure. Solvent: KOH in D_2_O, pD 11.4. Temperature: 20 °C. Black line is a fit of experimental data with a model of elliptical cylinder. Orange and brown lines are fits with a model of charged prolate ellipsoid. For better representation, scattering curves for ellipsoids are offset by factors of 0.3 (**a**) and 0.2 (**b**).

Let us compare the viscosity data with the variation in the structure of the system revealed by SANS. The viscosity dependences can be divided into three ranges, where the solutions have different properties (Figure 2b). For convenience, approximate concentrations of all the additives corresponding to the boundaries between those ranges are listed in Table 1. In the first range, at low additive concentrations, the viscosity drops only slightly (by less than an order of magnitude) and stays close to the initial viscosity of the solutions without additives and 10^3^–10^5^ times higher than the viscosity of water. In this range, the frequency dependences of the storage (G’) and loss (G”) moduli are characteristic of viscoelastic fluids (Figure 1c,d (210 mM AA); Figure S1 (44 or 62 mM NIPA); Figure S2 (12 mM 1-phenylhexane). It means that an entangled network of WLMs is preserved in the first range. At the same time, a slight viscosity decrease suggests that some amount of additive is solubilized inside the micelles. These observations coincide with SANS data (Figure 4a). Indeed, in the absence or at small additive concentrations, the scattering curves in a wide q range (0.04–0.45 Å^−1^) can be well fitted by a form-factor of a cylinder with elliptical cross-section and radii R_1_ = 16 Å and R_2_ = 25 Å (Table 2), R_1_ being close to the length of a fully extended potassium oleate tail (19 Å). It means that the local cylindrical geometry of WLMs is preserved. Note that even in the excess of the short-chain cationic surfactant (R = 1.5), which has a tail of ca. 9 Å, the radius of the micelles is determined by the long-chain anionic surfactant, which coincides with the data reported previously [61]. This is explained by partial segregation of anionic and cationic surfactants: oleate molecules with longer tails are preferentially located along the minor radius R_1_ of the elliptical cross-section, where the curvature is smaller and is optimal for them, while C8TAB species with shorter tails reside along the major radius R_2_, where the curvature is higher. At low q, the scattering curves deviate from the cylinder model due to the electrostatic interactions between the charged micelles and the volume excluded effects [62]. However, this does not affect the calculated radii of the WLMs cross-section, since R_1_ and R_2_ are determined from the high-q part of the scattering curve, and interactions affect mostly the low-q part [3,61]. However, when uncharged additive molecules are added, the low-q intensity further decreases. It might indicate the decrease in the micellar length. Some shortening of the micelles seems to be responsible for a slight drop of viscosity in the first range.

In the second range corresponding to intermediate additive concentrations, the viscosity drops sharply by 3–5 orders of magnitude down the water values (Figure 2b). This is accompanied by a disappearance of viscoelastic behavior (Figure 1, Figures S1 and S2), which indicates the disappearance of the entangled micellar network and transition to the unentangled semi-dilute regime for micellar chains [63]. In this regime, viscosity depends on the mean micellar length as η~L^1.9^ [64]. Therefore, the drop in viscosity and breaking of the network might be explained by a significant shortening of WLMs caused by solubilization of additives inside them. This is supported by a further decrease in the low-q SANS intensity in this range (Figure 4a).

Finally, in the third range corresponding to high additive concentrations, the viscosity stays nearly constant (1–3·10^−3^ Pa·s) and close to the viscosity of water. In this range, the SANS curves are fitted by a model of prolate ellipsoids (Figure 4), which means that WLMs are fully broken into small ellipsoidal aggregates that explain low viscosity of the solutions. Therefore, SANS data evidence that the addition of both hydrophobic and hydrophilic additives leads to the transformation of WLM network into small ellipsoidal aggregates.

Let us consider the structure of those aggregates for different additives under study. In the case of n-decane, previously it was shown [30] that the aggregates represent ellipsoidal microemulsion droplets with the following structure: a droplet of hydrocarbon is located in the center of the aggregate and is surrounded by a surfactant monolayer. At R = [C8TAB]/[oleate] = 1.5, the polar and equatorial radii are equal to 93 and 48 Å, respectively. The ellipsoidal form of microemulsion droplets (instead of spherical one) was explained by partial segregation of oleate from C8TAB at the surfactant monolayer which surrounds a hydrocarbon droplet. Oleate molecules with longer tails are believed to be located mainly at the ellipsoid equator, where the curvature is lower, and where the packing conditions are optimal for them, whereas C8TAB prefer to reside at the ellipsoid poles where the curvature is higher.

In the case of 1-phenylhexane, the aggregates also represent ellipsoidal microemulsion droplets. At R = 1.5, the polar and equatorial radii of those droplets are the following: R_pol_ = 98 Å, R_eq_ = 65 Å (Figure 4b). So, the microemulsion droplets with 1-phenylhexane are somewhat larger than those with n-decane. It may be explained by the fact that hydrocarbon with aromatic ring (1-phenylhexane) can partially penetrate in the surfactant shell of the microemulsion droplets due to π-cationic interaction with C8TAB molecules [65]. As a result, 1-phenylhexane resides both in the central part of the droplet and in the surfactant layer, which slightly increases the volume of the droplets as compared to n-decane, which is located only in the central part.

In the case of AA, the ellipsoidal aggregates have much smaller radii: R_pol_ = 40 Å, R_eq_ = 18.3 Å (Figure 4a). Their equatorial radius is close to the fully extended length of the oleate tail, which suggests that AA molecules do not form a droplet inside the aggregate but are located in the micellar corona. Previously, this was observed for micelles of a single surfactant (potassium oleate) with added AA [24]. The location of AA in the micellar corona increases the effective area per surfactant head a_e_, thereby diminishing the molecular packing parameter v_o_/a_e_l_o_ [66] (v_o_ and l_o_ are the volume and the length of the surfactant tail) and favoring the spherical packing of surfactant instead of cylindrical one. Therefore, in the case of hydrophobic additives (n-decane and 1-phenylhexane) the destruction of WLM network leads to the formation of ellipsoidal microemulsion droplets, whereas in the case of hydrophilic additives (NIPA and AA) the ellipsoidal micelles are formed.

Thus, in general, all the additives act similarly in breaking WLMs: they induce the shortening of the micelles, followed by breaking of the micellar network and transformation of WLMs into small ellipsoidal aggregates. However, much larger droplets of surfactants with added hydrocarbons rather than with hydrophilic additives show that the solubilization of these species proceeds differently in the surfactant aggregates. Moreover, the amounts of hydrophilic additives per surfactant necessary to break WLMs are much larger than the corresponding amounts of hydrocarbons (Table 1). This suggests that monomers and hydrocarbons have different “efficiency” in breaking WLMs and are differently distributed between surfactant aggregates and the solution. Therefore, studies of the aggregates after complete breaking of WLMs were performed.

### 3.3. Distribution of Additives

**Figure 5:**
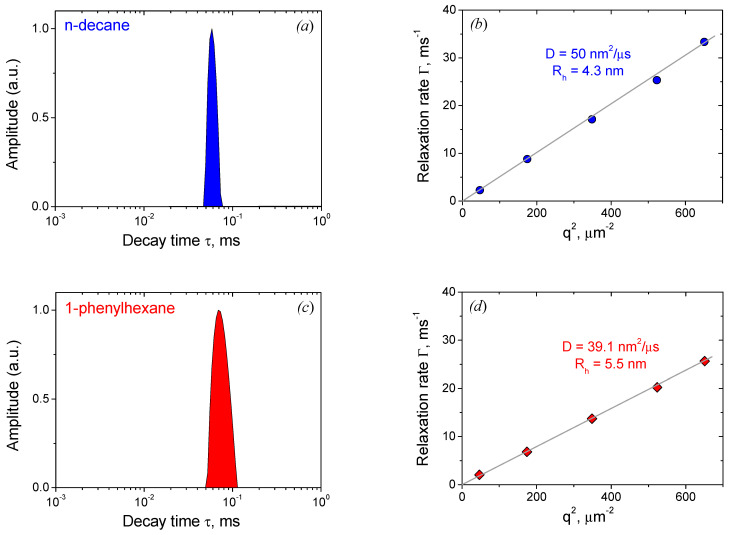

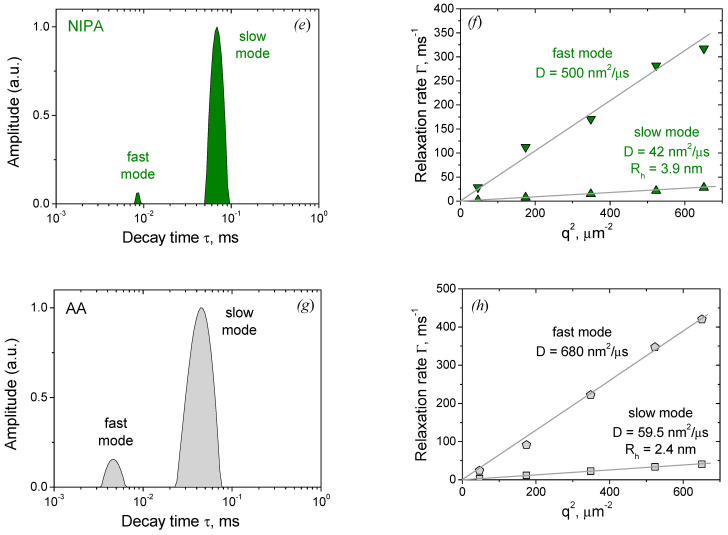
Decay time (τ) distributions (on the left) and dependences of relaxation rates (Γ) on the square of scattering vector q^2^ (on the right) obtained by DLS for the solutions of 78 mM potassium oleate, 117 mM C8TAB and various additives: (**a**,**b**) 210 mM n-decane; (**c**,**d**) 250 mM 1-phenylhexane; (**e**,**f**) 620 mM NIPA; (**g**,**h**) 2810 mM AA. Solvent: KOH in D_2_O, pH 11. Temperature: 20 °C.

Surfactant aggregates formed in dilute solutions at large concentrations of additives were studied by DLS (Figure 5a–h). For aggregates with absorbed hydrocarbons, distribution functions of the scattering objects on decay times (τ) are monomodal—they show one characteristic peak (Figure 5a,c). Relaxation rates Γ = 1/τ obey linear dependences on the square of the scattering vector q^2^ (Figure 5b,d), which means that the peaks are diffusive. The peaks correspond to the diffusion of microemulsion droplets formed in the presence of n-decane or 1-phenylhexane. From the slopes of the dependences Γ = Dq^2^, diffusion coefficients D of the droplets were determined, and their hydrodynamic radii R_h_ were calculated from Stokes–Einstein equation:

R_h_ = kT/6πη_s_D, (1)

where k is the Boltzmann constant, T the absolute temperature, and η_s_ the solvent viscosity. For n-decane and 1-phenylhexane, R_h_ values are equal to 4.3 and 5.5 nm, respectively (Table 3). The radii are 2.2–2.9 times larger than the length of fully extended oleate alkyl tail, which supports the fact that hydrocarbons are solubilized in the central part of the droplet. The radii determined from DLS are consistent with SANS data. For instance, for n-decane *R_pol_* = 48 Å, *R_eq_* = 93 Å [30], and thus the radius of gyration [67]

(2) $$R_{g}=\sqrt{\frac{2R_{eq}^{2}+R_{pol}^{2}}{5}}=51\;Å.$$

Therefore, ρ = *R_g_*/R_h_ = 1.2, which is close to the data published previously for ellipsoids (ρ values less than 2.24 were reported, and ρ should be larger than the value of 0.77 for spheres) [68].

**Figure 6:**
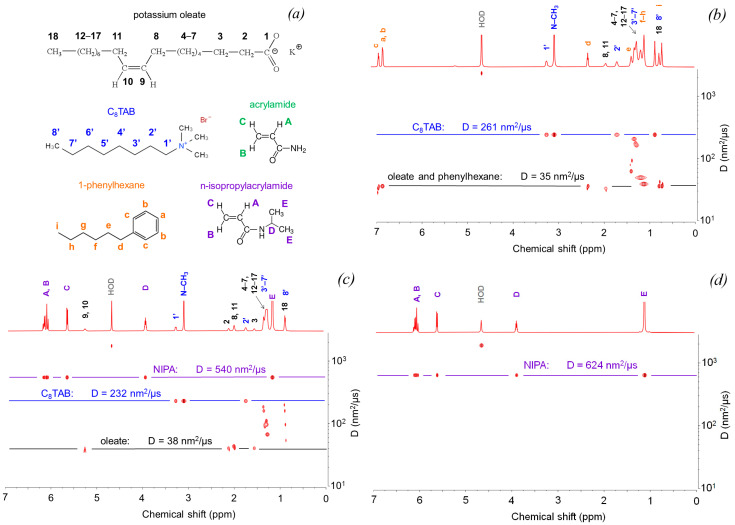

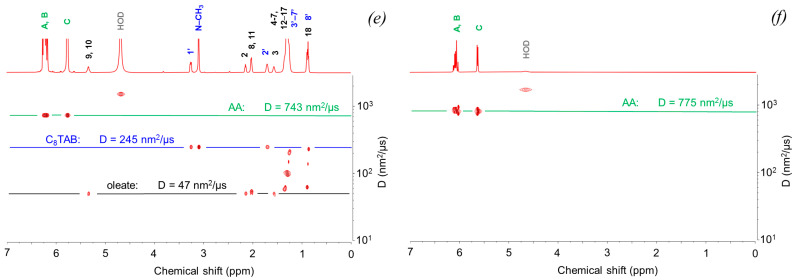
(**a**) Chemical structures of surfactants and additives. (**b**–**f**) Pseudo-2D DOSY spectra for different systems: (**b**) surfactants with 250 mM 1-phenylhexane; (**c**) surfactants with 620 mM NIPA; (**d**) 620 mM NIPA without surfactants; (**e**) surfactants with 20 wt% AA; (**f**) 20 wt% AA without surfactants. Concentrations of surfactants: 78 mM potassium oleate, 117 mM C8TAB. Solvent: KOH in D_2_O, pH 11.

Since hydrocarbons are highly hydrophobic and have a very low water solubility (see above), it is expected that all the hydrocarbon molecules are solubilized inside the surfactant aggregates. For 1-phenylhexane, this is proven by DOSY (Figure 6). Figure 6b shows a typical pseudo-2D DOSY plot for C8TAB/oleate aggregates with solubilized 1-phenylhexane. Diffusion coefficient corresponding to potassium oleate groups (C9,C10 methine protons; C3,C8,C11 and some other methylene protons, and C18 methyl protons) is equal to 35 nm^2^/μs. From the Stokes–Einstein equation, taking into account higher solvent viscosity in DOSY NMR measurements than in DLS (for DOSY, D_2_O with η_s_ = 1.25 mPa·s [69] is used), the value of R_h_ = 5.1 nm is calculated, which is very close to the hydrodynamic radius of the microemulsion droplets determined by DLS (Table 2). Therefore, the diffusion coefficient of the oleate species coincides with those of the surfactant aggregates. This is reasonable since potassium oleate has a long hydrophobic tail and exhibits strong hydrophobic interactions within the micelle, leading to slow exchange of oleate species between the micelles, and to a very low fraction of free oleate not included in the micelles (critical micelle concentration of potassium oleate is as low as ~0.9 mM [70] and decreases to ca. 0.2 mM when mixed with C8TAB [71], which is much less than its concentration in the present work—78 mM).

From Figure 6b, it is seen that the diffusion coefficient corresponding to all peaks of 1-phenylhexane (protons of the benzene ring and of the alkyl tail) is equal to those of the surfactant aggregates. This indicates that all the hydrocarbon molecules are located inside the droplets, e.g., its partition coefficient (fraction of molecules included in surfactant aggregates) *p* = 1.

**Figure 7:**
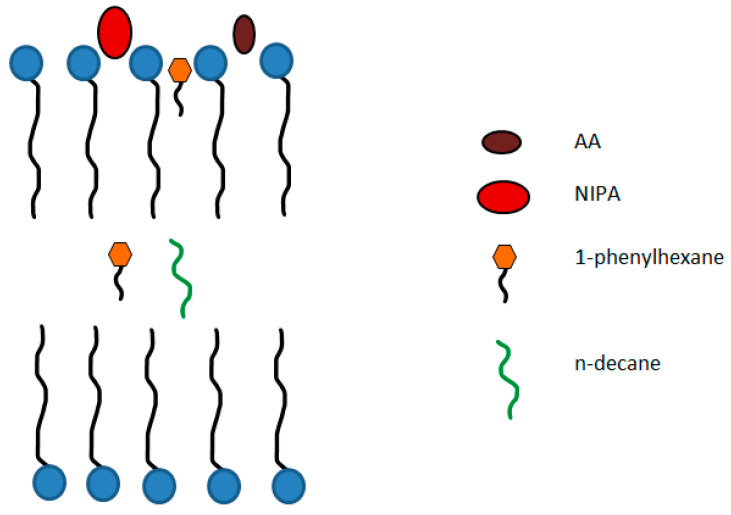
Schematic representation of the location of different additives inside the surfactant aggregates.

For n-decane, it was not possible to determine its diffusion coefficient from DOSY data due to overlapping of hydrocarbon and surfactant signals in the ^1^H NMR spectrum. However, it is most probable that all n-decane molecules reside inside the micelles as for 1-phenylhexane, since the n-decane is even more hydrophobic than the 1-phenylhexane. Since both hydrocarbons are completely solubilized within the micelles, what is the reason for the shift of viscosity curve to higher concentration of added hydrocarbon, when 1-phenylhexane is used instead of n-decane (Figure 2a)? Most obviously, this is due to the aromatic ring, which favors the solubilization of the hydrocarbon at or near the core/water interface (Figure 7) as a result of the π-cationic interaction with the head groups of C8TAB [69]. When the absorbed hydrocarbon resides near the core/water interface, the viscosity does not change considerably. Only when the outer layer becomes saturated with 1-phenylhexane, further portions of 1-phenylhexane penetrate inside the core (Figure 7) leading to a decrease in viscosity [13].

From Figure 6, it is seen that the diffusion coefficient of C8TAB molecules determined by DOSY NMR is higher than those of the surfactant aggregates: D = 261 nm^2^/μs. According to the literature [72,73], this may indicate that some fraction of C8TAB is included in the micelles, whereas some fraction of C8TAB is residing in water. Fast exchange between these two species, probably arising from short length of the C8TAB alkyl tail and weak hydrophobic interactions, results in registering one single apparent value of the diffusion coefficient D_app_ being a weighted average of the diffusion coefficient of free C8TAB species (D_free_) and diffusion coefficient of the surfactant aggregates D_agg_ [74]:

D_app_ = *p* × D_agg_ + (1 − *p*) × D_free_. (3)

A value of D_free_ = 680 nm^2^/μs was obtained by DOSY for C8TAB molecules (Figure S5) in the absence of oleate and below the CMC. It equals to 0.29 M [75]. Therefore, one can estimate that the partition coefficient p_C8TAB_ = 0.65, that is, only 2/3 of C8TAB molecules are included in the micelles. This is an interesting result showing that the molar ratio C8TAB/oleate in the micelles is nearly 1 despite the initial molar ratio R = 1.5 (R·p_C8TAB_ = 1). This means that oleate aggregates can accumulate only equimolar amount of the oppositely charged surfactant, while the other C8TAB molecules stay in the solution.

Note that there are some additional peaks in the region of methylene protons (Figure 6b) with diffusion coefficients, intermediate between those of C8TAB and oleate. These are well-known artefacts of DOSY spectral processing caused by overlap of the signals with different diffusion coefficients [76]. While it is possible to resolve them using multivariate algorithms [77] or advanced 3D NMR pulse sequences [78], the reliability of such approaches is still lower than determining the diffusion coefficient values from the signals that show no spectral overlap, as shown be horizontal lines on Figure 6.

Let us now consider the mixtures of WLMs with hydrophilic additives. For AA and NIPA, distribution functions of the scattering objects on decay times (τ) are bimodal (Figure 5e,g). Dependences Γ(q^2^) are linear and pass through the origin for all peaks, showing that the peaks are diffusive (Figure 5f,h). In order to attribute the modes at these distributions, DLS results were compared to DOSY measurements (Figure 6c,e). For both cases, diffusion coefficients of oleate are of the same order of magnitude as for the case of 1-phenylhexane. This suggests that oleate diffusion coefficient coincides again with D of the surfactant aggregates. Moreover, R_h_ values calculated from oleate DOSY peaks perfectly coincide with R_h_ values of the slow mode at the DLS distributions (see Table 2; values of the solvent viscosity η_s_ for calculation of R_h_ were taken from Figure S4 with consideration that the solvent contains free monomers, see below). This means that the slow mode corresponds to the surfactant aggregates with solubilized hydrophilic additives—monomers.

However, contrary to the case of 1-phenylhexane, the diffusion coefficients of NIPA and AA determined from DOSY differ significantly from those of the surfactant aggregates and are closer to D values for free NIPA and AA in the absence of surfactants (Figure 6d,f). This means that the monomers are distributed between the micelles and the solution, and fast exchange between these two species proceeds. Using the Equation (3), the fractions of NIPA and AA in the micelles were found to be only 0.14 and 0.04, respectively. Therefore, due to their high water solubility, most of the hydrophilic monomer molecules reside in the solution, and only a small portion of them penetrates into the micelles. More hydrophobic NIPA has a higher value of *p*. The interaction of hydrophilic monomers with the micelles can be due to the presence of some hydrophobic fragments in the monomers: CH_2_=CH and isopropyl groups in NIPA and CH_2_=CH group in AA. Higher number of hydrophobic fragments in NIPA is responsible for larger fraction of NIPA solubilized in the micelles. If the distribution of monomers between micelles and solution is taken into account, and viscosity is plotted as a function of relative volume of the additive in the micelles (*p*·(vol. additive)/(vol. surfactants), Figure S3b), the curves for AA and NIPA coincide. Therefore, the shift of the viscosity dependence on the additive concentration for AA relative to NIPA (Figure 2a) is related solely to the higher fraction of NIPA molecules interacting with the micelles. At the same time, mechanism of WLM breaking by these two monomers is the same.

Based on the DOSY results, one can assume that the fast modes at the DLS distributions correspond to free monomers. Indeed, DOSY and DLS produce close values of D, though DLS values are slightly underestimated. In similar DLS experiments, small individual molecules were previously detected, for instance, mono- and disaccharides [79,80,81] or even smaller molecule 4-(3-chloropropoxy)phenol [82].

Therefore, free water-soluble monomers co-exist with surfactant aggregates in which some fraction of monomers is penetrated. The hydrodynamic radii of the aggregates are significantly smaller than for n-decane and 1-phenylhexane, and they decrease when the monomer concentration increases down to the values R_h_ = 2.3 nm (at 970 mM NIPA) and R_h_ = 1.9 nm (at 4220 mM AA). Therefore, DLS and DOSY data support the hypothesis that hydrophobic and hydrophilic additives penetrate differently in the surfactant aggregates: hydrophobic species (n-decane and 1-phenylhexane) are mainly located as droplets deep inside the micellar cores or at the core/corona interface, whereas the hydrophilic species (AA and NIPA) reside at the micellar corona area (Figure 7).

**Figure 8:**
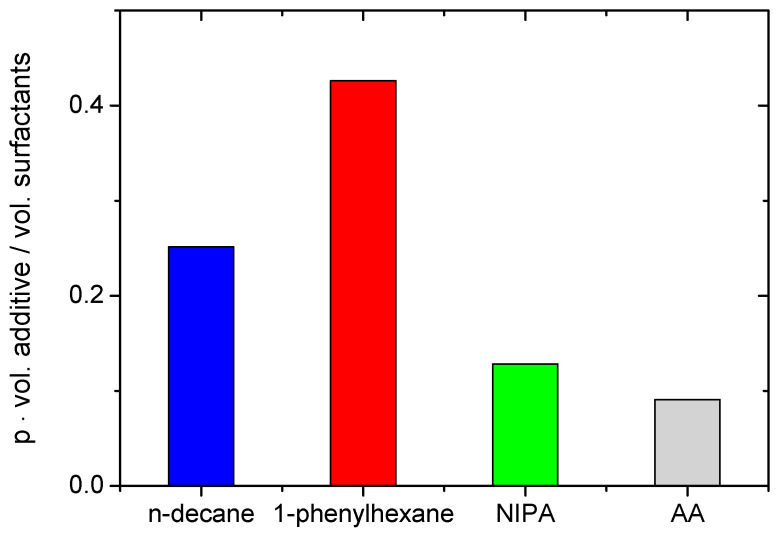
Relative volumes of various additives (v_rel_ = *p*·vol. additive/vol. surfactant) inside surfactant aggregates corresponding to the minimum additive concentration necessary to reach minimum viscosity (corresponding to the cross-over from Range 2 to Range 3, see Table 1).

In order to generalize the effect of various additives on WLMs, relative volumes of additives inside the aggregates (taking into account the partition coefficients p: v_rel_ = p·vol. additive/vol. surfactant) which are necessary to disrupt WLMs can be compared (Figure 8). For all additives, they are in the range 0.1–0.45, meaning that ca. 9–30% of the droplet’s volume is occupied by the additive, while the other is left for surfactant. The values of v_rel_ are slightly higher for hydrocarbons than for monomers, probably due to the fact that they form larger aggregates. The highest v_rel_ is seen for 1-phenylhexane, which may be a result of two reasons. First, 1-phenylhexane forms the largest droplets. If one suggests that the droplet is covered by a surfactant monolayer with a constant thickness, the volume of the hydrocarbon core inside the droplet should roughly increase as ~R_h_^3^ with the droplet radius R_h_, while the volume of the surrounding surfactant layer is proportional to ~R_h_^2^. This means that the ratio of core and shell volumes (e.g., v_rel_) increases with the droplet size. The second reason might be some penetration of 1-phenylhexane molecules into the surfactant layer, which may be possible due to the presence of a benzene ring able to interact with cationic polar head of C8TAB [65]. This additionally increases the amount of hydrocarbon in the droplet and decreases the relative volume of surfactants in the shell.

## 4. Conclusions

Thus, the breaking of wormlike surfactant micelles induced by additives of different hydrophobicity (water-insoluble hydrocarbons n-decane and 1-phenylhexane and water-soluble monomers NIPA and AA) was investigated. It was shown that hydrophobic additives destroy the WLMs more effectively. It may be due to two main reasons: (i) they are completely solubilized inside the micelles, and (ii) they are accommodated mainly in the micellar core, which favors WLM destruction more efficiently than the solubilization in the corona area or core/corona interface.

Among the two hydrophobic additives, 1-phenylhexane is somewhat less efficient most probably because it is partially located near the core/corona interface (Figure 7) due to π-cation interactions with C8TAB heads. When the corona becomes saturated with 1-phenylhexane, further portions of 1-phenylhexane penetrate deep into the core (Figure 7) forming a hydrocarbon droplet [13]. As a result, higher amount of aromatic hydrocarbon is required to induce the transformation of WLMs into microemulsion.

As to water-soluble monomers, one needs to add them in very large amounts to destroy the WLMs. This is because only a small portion of those compounds penetrate the micelles (14% of NIPA and 0.04% of AA). Moreover, they are localized in the corona area (Figure 7) and do not penetrate deep into the micellar interior. Such location increases the effective area per surfactant head, thereby favoring the spherical packing of surfactant instead of cylindrical one. As a result, in the case of hydrophilic additives, ellipsoidal micelles are formed.

Despite different kinds of species formed as a result of solubilization of hydrophobic and hydrophilic additives inside the WLMs, the evolution of the viscosity upon destruction of WLMs in both cases is quite similar. Moreover, the viscosity vs. additive volume fraction curves can be even perfectly superimposed by an appropriate shift along the volume fraction axis. It indicates that the transformation of WLMs into the microemulsion (in the case of hydrophobic additives) or into the ellipsoidal micelles (in the case of hydrophilic additives) proceeds in a similar way.

The results obtained contribute to the fundamental understanding of the transformations of wormlike surfactant micelles upon absorption of hydrophobic and hydrophilic moieties and provide insight into the opportunities for manipulating their structure and properties and optimizing their performance in practical applications including oil industry (hydraulic fracturing), synthesis of amphiphilic copolymers in micellar media, and so forth.

## Figures and Tables

**Table 1:** Approximate additive concentrations (mM) corresponding to different ranges of viscosity drop in Figure 2b.

	Crossover from Range 1 to Range 2			Crossover from Range 2 to Range 3		
Type of Additive	Additive, mM		Additive / Surfactants vol./vol.	Additive, mM		Additive / Surfactants vol./vol.
	R = 0.5	R = 1.5		R = 0.5	R = 1.5	
n-decane	7	10.5	0.04	35	70	0.22
1-phenylhexane	12.6	21	0.08	77	123	0.44
NIPA	44	73	0.18	264	440	1
AA	210	314	0.4	1125	1970	2.2

**Table 2:** Fitting parameters of SANS curves obtained from aqueous solutions containing 78 mM potassium oleate, 117 mM C8TAB and different additives (Figure 4).

Type of Additive	Concentration of Additive, mM	SANS Model	R_eq_ (R_1_), Å	R_eq_ (R_1_) Poly- Dispersity	R_pol_ (R_2_), Å	R_pol_ (R_2_) Poly- Dispersity	Charge, e
-	0	elliptical cylinder	16	0.05	25	0.05	-
1-phenylhexane	250	charged prolateellipsoid	65	0.12	98	0.1	40
AA	4220	charged prolateellipsoid	18.4	0.1	45	0.15	15

**Table 3:** Approximate additive concentrations (mM) corresponding to different ranges of viscosity drop in Figure 2b.

Additive	Concentration, mM	DLS			DOSY			*p*
		D, nm^2^/μs	η_s_, * mPa·s	R_h_, nm	D, nm^2^/μs	η_s_, * mPa·s	R_h_, nm	
n-decane	210	50	1	4.3	-	-	-	1 **
1-phenylhexane	250	39.1	1	5.5	35	1.25	5.1	1
NIPA	620	42	1.3	3.9	38	1.56	3.6	0.14
	970	60.7	1.54	2.3	-	-	-	-
AA	2810	59.5	1.5	2.4	47	1.8	2.5	0.04
	4220	63.6	1.82	1.9	-	-	-	-

* Viscosities of solutions of the additives in H_2_O (for DLS) or in D_2_O (for DOSY) taken from Figure S4. ** Partitioning coefficient of n-decane is considered to be unity based on its higher hydrophobicity than of 1-phenylhexane.

## Data Availability

Data are available from the authors upon reasonable request.

## Supplementary Materials

The following supporting information can be downloaded at: https://www.mdpi.com/article/10.3390/nano12244445/s1, Figure S1: Frequency dependences of the storage modulus G’ (filled symbols) and loss modulus G’’ (open symbols) for aqueous solutions containing 78 mM potassium oleate, 39 mM (a) or 117 mM (b) C8TAB, and different concentrations of NIPA indicated in the Figure: 0 mM (squares), 9 mM (circles), 44 or 62 mM (tiangles), 88 mM (diamonds). Temperature: 200C, pH 11. Figure S2: Frequency dependences of the storage modulus G’ (filled symbols) and loss modulus G’’ (open symbols) for aqueous solutions containing 78 mM potassium oleate, 39 mM (a) or 117 mM (b) C8TAB and different concentrations of 1-phenylhexane indicated in the Figure: 0 mM (squares), 12 mM (circles), 18 mM (tiangles), 25 mM (diamonds). Temperature: 200C, pH 11. Figure S3: Dependences of zero-shear viscosity on the molar ratio β ·[additive]/[surfactant] (a) or on the volume ratio *p*·(vol. additive)/(vol. surfactant) (b) for aqueous solutions containing 78 mM potas-sium oleate, 39 mM (filled symbols) or 117 mM (open symbols) C8TAB and different additives: AA (squares), NIPA (triangles), 1-phenylhexane (diamonds), n-decane (circles). β are arbitrary shift factors, and *p* are partitining coefficients determined form DOSY data (Table 2). Figure S4: Dependences of relative viscosity on monomer concentration for solutions of AA in H_2_O (circles), AA in D_2_O (triangles), NIPA in H_2_O (diamonds), NIPA in D_2_O (squares). Temperature 20^0^C. Figure S5: Pseudo-2D DOSY spectrum for 39 mM C8TAB solution in D2O, pH 11.
