# Supplementary material for: Universal Character of Breaking of Wormlike Surfactant Micelles by Additives of Different Hydrophobicity
Source: Nanomaterials (Basel). 2022 Dec 14;12(24):4445. doi: 10.3390/nano12244445 (PMC9781539; doi:10.3390/nano12244445)
Supplement: Supplementary file 1 [file nanomaterials-12-04445-s001.zip › nanomaterials-2060298-supplementary.pdf]

nanomaterials

MDPI

Article

Universal character of breaking of wormlike surfactant micelles by additives of different hydrophobicity

Andrey V. Shibaev[1,*], Alexander S. Ospennikov[1], Elizaveta K. Kuznetsova[1], Alexander I. Kuklin[2,3], Teimur M. Aliev[4], Valentin V. Novikov[3] and Olga E. Philippova[1]

[1] Physics Department, Moscow State University, 119991 Moscow, Russia
[2] Frank Laboratory of Neutron Physics, Joint Institute for Nuclear Research, 141980 Dubna, Russia
[3] Moscow Institute of Physics and Technology, 141701 Dolgoprudny, Russia
[4] A. N. Nesmeyanov Institute of Organoelement Compounds, Russian Academy of Sciences, 119991 Moscow, Russia
* Correspondence: shibaev@polly.phys.msu.ru; phil@polly.phys.msu.ru; Tel.: +7(495)939-14-64

Supplementary materials

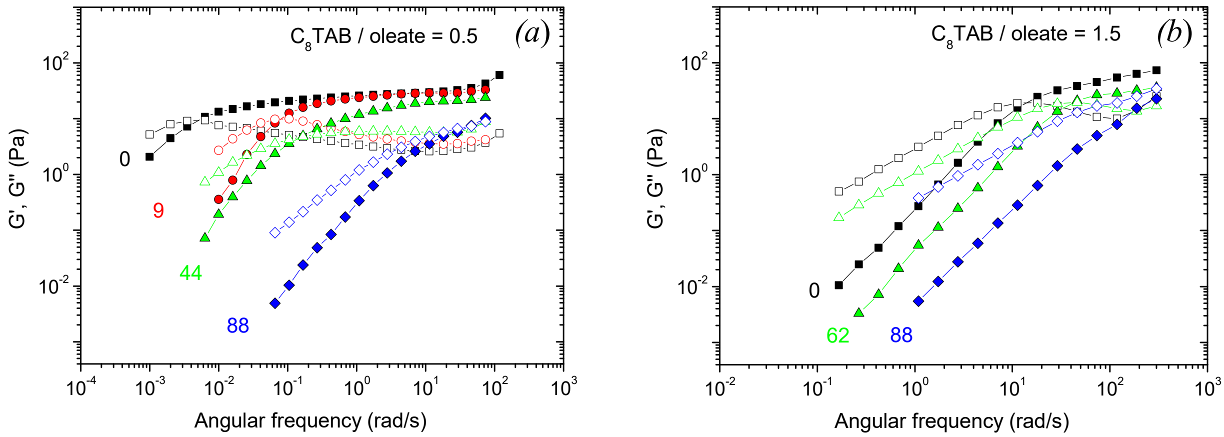

Figure S1. Frequency dependences of the storage modulus G′ (filled symbols) and loss modulus G′′ (open symbols) for aqueous solutions containing 78 mM potassium oleate, 39 mM (a) or 117 mM (b) C8TAB, and different concentrations of NIPA indicated in the Figure: 0 mM (squares), 9 mM (circles), 44 or 62 mM (tiangles), 88 mM (diamonds). Temperature: 20^{0}C, pH 11.

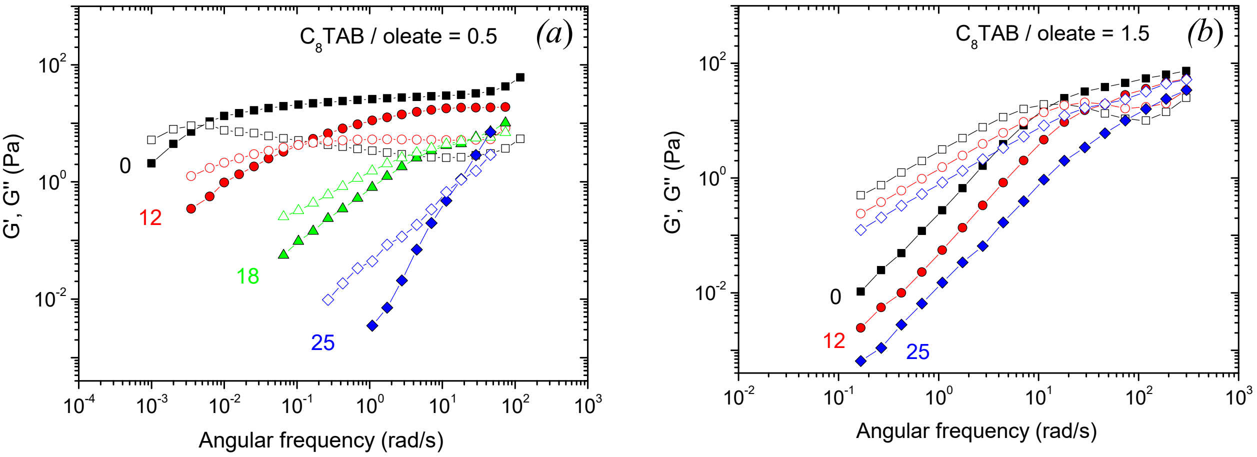

Figure S2. Frequency dependences of the storage modulus G′ (filled symbols) and loss modulus G′′ (open symbols) for aqueous solutions containing 78 mM potassium oleate, 39 mM (a) or 117 mM (b) C8TAB and different concentrations of 1-phenylhexane indicated in the Figure: 0 mM (squares), 12 mM (circles), 18 mM (tiangles), 25 mM (diamonds). Temperature: 20^0C, pH 11.

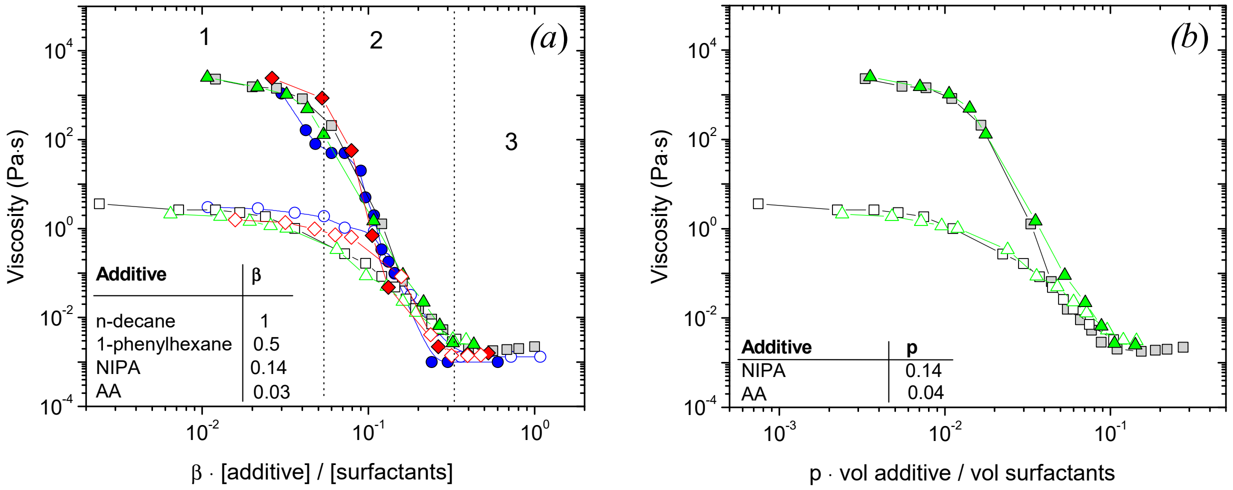

Figure S3. Dependences of zero-shear viscosity on the molar ratio β·[additive]/[surfactant] (a) or on the volume ratio p·(vol. additive)/(vol. surfactant) (b) for aqueous solutions containing 78 mM potassium oleate, 39 mM (filled symbols) or 117 mM (open symbols) C8TAB and different additives: AA (squares), NIPA (triangles), 1-phenylhexane (diamonds), n-decane (circles). β are arbitrary shift factors, and p are partitining coefficients determined form DOSY data (Table 2).

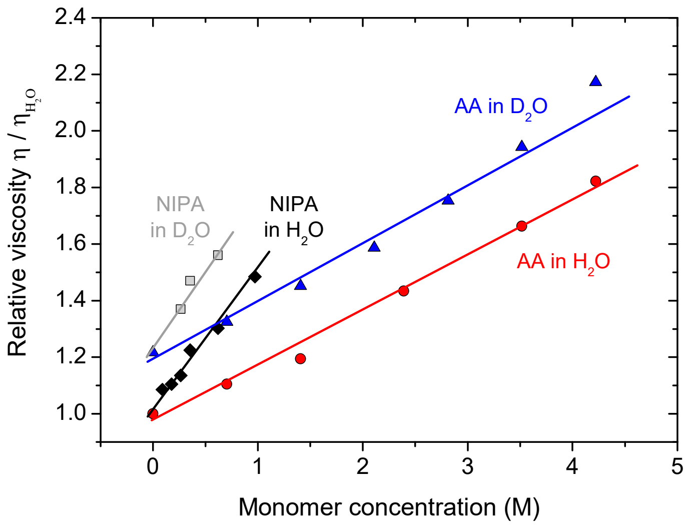

Figure S4. Dependences of relative viscosity on monomer concentration for solutions of AA in H_2O (circles), AA in D_2O (triangles), NIPA in H_2O (diamonds), NIPA in D_2O (squares). Temperature 20^{0}C.

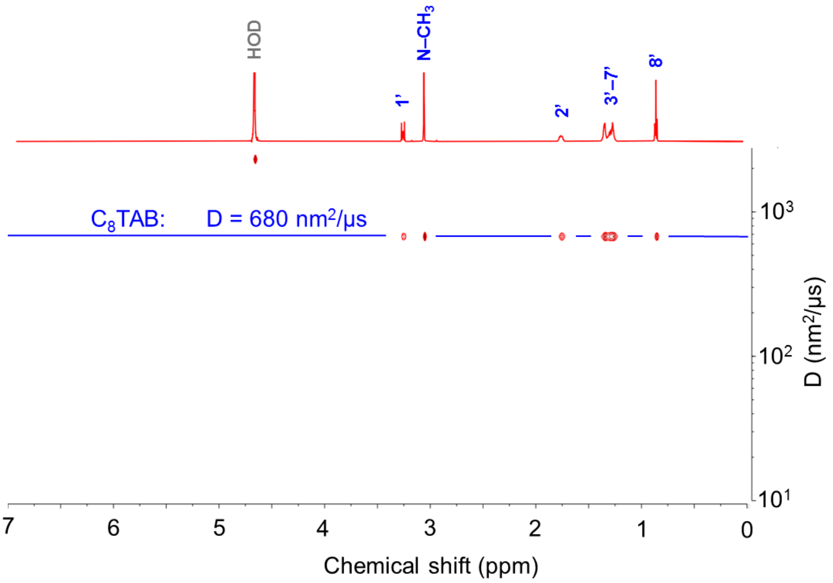

Figure S5. Pseudo-2D DOSY spectrum for 39 mM C8TAB solution in D_2O, pH 11.
